# (Mis)measuring men’s involvement in global health: the case of expectant fathers in Dakar, Senegal

**DOI:** 10.1186/s12884-022-05093-0

**Published:** 2022-10-07

**Authors:** Richard Powis

**Affiliations:** College of Public Health, University of South Florida, 13201 Bruce B Downs Blvd, Tampa, FL 33612 USA

**Keywords:** Maternal and child health, Maternal support, Men’s involvement, Therapy management groups, Gender and health

## Abstract

**Background:**

In 2018, USAID published a report based on Demographic and Health Surveys data on the relationship between men’s involvement and women and children’s health outcomes. Using a flawed operationalization of “men’s involvement,” USAID’s analysis implies that Senegalese men are not involved in women and children’s health.

**Methods:**

The findings of this study come from 12 months of ethnographic research in Dakar, Senegal that examined the roles and responsibilities of expectant fathers. Research participants included 32 pregnant women and 27 expectant partners recruited from three maternity wards. Research methods included long-term, immersive participant observation and semi-structured interviews.

**Results:**

Pregnant women in Senegal are surrounded by a kin-based network of care providers called the *entourage* who share responsibilities for support. Expectant fathers, as members of the entourage, are expected to provide financial and emotional support, while other members of the entourage are expected to undertake the responsibilities which USAID have designated as “men’s involvement.” Men typically do not undertake additional forms of care and support because they are considered “women’s business,” meaning that women actively discourage men from doing those things, in order to preserve women’s autonomy.

**Conclusion:**

This research demonstrates that expectant fathers are involved in antenatal care in ways that USAID does not track through DHS. Further, I argue that USAID’s heterosexist, monogamous, and nuclear operationalization of “men’s involvement” aligns with a long history of Eurocentrism in development discourse which may be potentially harmful and obstructive to improving maternal and child health when the problem that is targeted is not a problem at all. This study is yet another case that demonstrates an urgent need of public and global health engagement with local stakeholders and ethnographic researchers.

**Supplementary Information:**

The online version contains supplementary material available at 10.1186/s12884-022-05093-0.

## Background

In 2010, Lu and colleagues asked, “Where is the F in MCH?” [1] referring to research on father involvement in Maternal and Child Health (MCH). More than ten years later, there is much research about the influences that fathers have on child health and development [2]. Researchers have found that fathers have positive impacts on children’s cognitive development [3–5], peer relationships, risky behaviors, and decreased odds of incarceration [6–11]. In 2019, the National Home Visiting Resource Center (NHVRC) noted that father involvement supports attachment and promotes children’s emotional regulation [12]. With respect to male involvement in home visiting programs, fathers self-report “improved knowledge of child development and positive parenting,” “better anger management,” and “greater connects to employment, educational opportunities, and other community services” [12]. In a systematic review, Tokhi and colleagues found that men’s involvement interventions have mostly positive effects on care-seeking, couple relationship dynamics, and home care practices [13]. The authors point out that while interventions may produce these positive effects, it remains to be seen whether these effects are mechanisms of reducing maternal morbidity or mortality.

In 2018, the United States Agency for International Development (USAID) released a report titled “Does Men’s Involvement Improve the Health Outcomes of their Partners and Children?” (henceforth “DHS 64”). In order answer their question, Assaf and Davis, the authors, draw on a well-known model from health promotion, “knowledge-attitude-behavior” [14] (Table 1). In the report, “men’s involvement” is defined using three independent variables: “correct knowledge, positive attitudes, and supportive behaviors toward the health of their partners and children” [15]. While the authors do not define knowledge, attitudes, or behaviors, the variables are operationalized and derived from the most recently available Demographic and Health Surveys (DHS) data from 33 countries as follows:

**Table 1:** Independent variables (Assaf and Davis 2018)

Independent Variable	DHS-derived Indicator
**Knowledge**	Correct knowledge of fertile period
	Correct knowledge of fluid intake for child with diarrhea
**Attitude**	Agreement with, “Contraception is a woman’s business and a man should not have to worry about it”
	Agreement with “Women who use contraception may become promiscuous”
**Behavior**	Present during ANC visit
	Discussed family planning with a healthcare worker
	Joint decision on how to spend men’s earnings

These independent variables were compared to the following outcome variables:

- unmet need for family planning
- contraceptive prevalence for modern methods
- four or more visits for antenatal care
- first ANC visit before four months pregnancy
- delivery in a health care facility
- completing three doses of DPT vaccine
- treatment for diarrhea

Overall, with respect to Senegal, Assaf and Davis found the following: In the case of correct knowledge, 39.1% of surveyed Senegalese men knew to give water to a child with diarrhea while only 10.7% had correct knowledge of women’s fertile period. In the case of the positive attitudes, Senegalese men do not generally agree that contraception is only a woman’s business (88.9%), nor that using contraception leads one to become promiscuous (67.5%). In the case of supportive behaviors, Senegalese men were among the least likely to be present during an ANC check-up (20.6%) or discuss family planning with health workers (5.6%). Senegalese men ranked fairly low (27^th^) among those who decide with their partners how to spend men’s earnings (19.3%). The implication of these findings is that Senegalese men are uninvolved in women’s health, although DHS 64 was not conclusive that men’s involvement was causative of women’s and children’s health outcomes.

In this article, I argue that Assaf and Davis’ findings (i.e., that Senegalese men are uninvolved) are both true and deeply flawed. My long-term ethnographic study demonstrates that Senegalese men are in fact a key component of antenatal care, and that Assaf and Davis’ findings are limited by the operationalization of “men’s involvement” using a Eurocentric, biomedicalized set of indicators that privilege positivist measurement. After a brief explanation of how long-term, ethnographic methods provide a unique window into the lives of Senegalese expectant parents, I will discuss the cultural context of the *entourage* and gendered expectations therein. I will then explain what it is Senegalese men are expected to do and what they are not expected to do. In the discussion, I build on the work of Luis Avilés’ 2001 critical discourse analysis [16] of a 1994 USAID report, *Epidemiological Profile* of El Salvador to argue that development discourse which emphasizes Eurocentric metrics are still present almost 25 years later. I will highlight that the care and support activities men are expected by their families and communities to do are unseen by instruments like the DHS, while the things they are not expected to do are measured by those same instruments. This occlusion of local contexts results in studies such as DHS 64 which find men to be uninvolved when, in actuality, they are involved. I will argue that such formulations of family dynamics and, in particular, masculinities only serve to perpetuate harmful stereotypes of men in LMIC. I conclude that this case is yet another example of the urgent need for global and public health engagement with local stakeholders and ethnographic researchers who can assist in the development of context-dependent and culturally appropriate metrics, program objectives, and goals.

## Methodology

The findings of this article come from an ethnographic study conducted in Dakar, Senegal over 12 months in 2018. The purpose of the study was to examine the ways that expectant fathers involve themselves in antenatal care, what they are expected or encouraged to do by their partners, families, social networks, and the State, and how they navigate these activities in light of the social expectations of masculinity. The findings of this study come from data collection methods including semi-structured interviews [17, 18] and participant-observation [19]. All interviews were conducted in Wolof, Peul, or French, transcribed in French by the author or the two research assistants, and thematically team-coded using MAXQDA12 [20].

### Recruitment and participants

In Senegal, pregnancy is both taboo and highly gendered and makes recruitment of expectant fathers particularly challenging. It is taboo in that talking about a specific pregnancy can attract bad luck or misfortune. Americans have a similar taboo that typically lasts for the first trimester of pregnancy, but in Senegal this taboo holds for the entire pregnancy. In practice, what this means is that all people who know of a particular pregnancy (e.g. family members, expectant fathers) should not speak to outsiders about it. It is also highly gendered, meaning that men typically find the topic embarrassing to talk about. With both of these cultural aspects combined, locating men who are willing to (a) identify themselves as expectant fathers and (b) talk about pregnancy can be difficult.

To overcome this challenge, the author worked closely with midwives and chief medical officers in three hospitals across the city of Dakar to develop a protocol in which pregnant women were recruited, interviewed, and then asked if their partners would be willing to be interviewed. Ultimately, we interviewed 32 pregnant women and 27 expectant fathers recruited evenly (i.e., about ten) from each hospital maternity ward.

### Semi-structured interviews

Semi-Structured Interviews lasted approximately one hour and took place either in maternity wards (when midwives could spare a room) or in a location of the interviewee’s choice (e.g. their home, workplace, a café). To avoid flouting the pregnancy taboo, we did not interview pregnant participants about their pregnancies directly (unless they volunteered information), rather we were interested in the experiences of pregnancy, who was caring for them, from whom they sought advice, and their relationships with their partners (Supplementary File 1). We asked similar questions of expectant fathers, e.g., how they were adjusting to the pregnancy, who was caring for their pregnant partners, from whom they sought advice, and what they were doing for their pregnant partners (Supplementary File 2).

### Participant-observation

Ethnographic research, as a kind of qualitative research, is unique in that it tends to be long-term and immersive for researchers. In addition to conventional qualitative data collection methods (e.g., focus group interviews, semi-structured interviews), a key method in the ethnographic toolkit is participant-observation. In general, participant-observation entails that the researcher eschews strong notions of objectivity, steps outside of the interviewer-interviewee relationship, and becomes an active participant in the phenomenon they are studying. This role of the researcher, to be present and attentive in the lives of research participants, affords opportunities and perspectives that can fill gaps in standard interview-based qualitative research – gaps that the researcher may not have even anticipated during the research design phase. This is a driving force of the iterative and ongoing qualities of ethnographic research.

The advantage of participant-observation is that the researcher is always “on” and therefore everything is “data.” This means that every social transaction, community or family meeting, or casual conversation (i.e. “unstructured interview”) can be counted as data collection (with ethical discretion, of course [21]). The obvious limitation of participant-observation is that under the conditions in which objectivity is less important than documenting lived experiences and possibilities, biases are likely to be introduced. Anthropologists control for these biases by, among other things, regular check-ins with community members and supplementing their research design protocols with additional methods.

With respect to this study, the author spent many hours over 12 months with expectant fathers in their daily lives – at work, running errands, family meals, religious holidays, doctor’s appointments, family celebrations (e.g., naming ceremonies), and more. Notably, the author was often present with couples and their families when pregnant participants were in labor and childbirth. While the findings of this article come from ethnographic research conducted in 2018, much of the cultural context (e.g., the *entourage*, gender roles) comes from a patchwork of exploratory visits to Dakar, Senegal over 10 months across the six preceding years in which the author sustained ongoing research relationships which included participant-observation and semi-structured interviews with young men and expectant fathers.

## Results

### The entourage

**Fig. 1:**
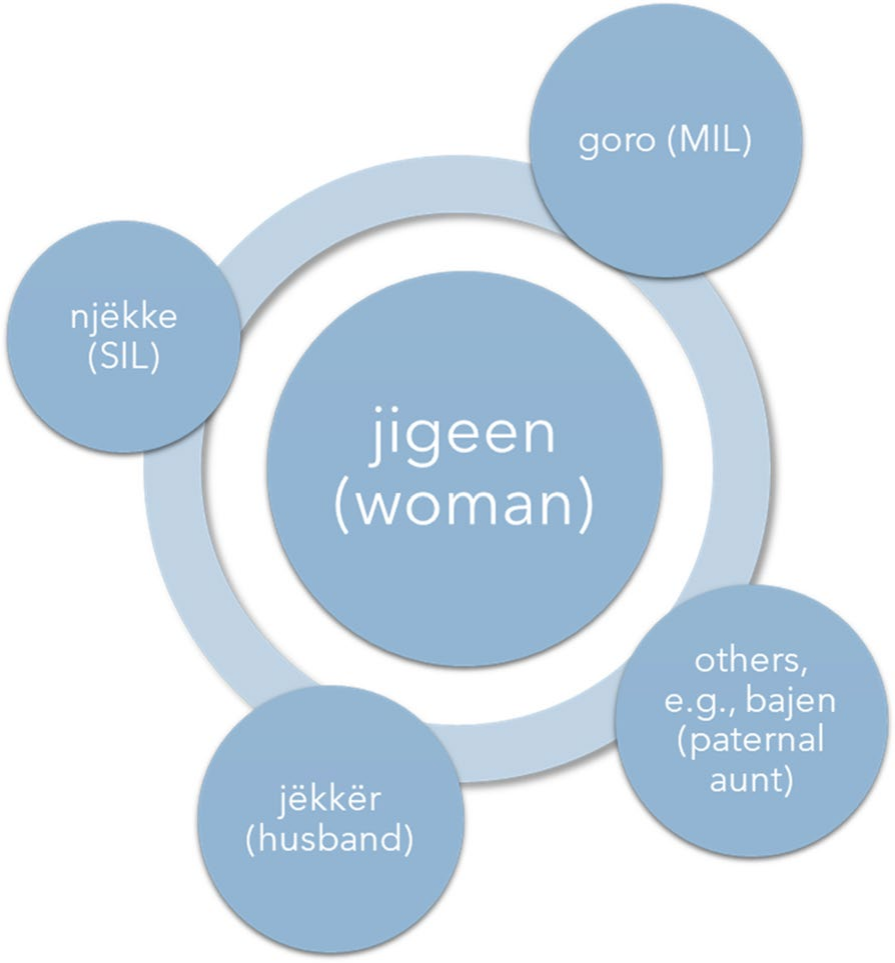
The Wolof entourag

The ideal situation in Dakar, in keeping with traditional Wolof kinship practices, is that pregnant women are married and that married women move into their husband’s household (which anthropologists call “virilocality”) [22]. It is in the home of their in-laws where pregnant women find the *entourage* (Fr. “people who surround”). The entourage is usually made up of their *jëkker* (husband), *goro* (mother-in-law), and *njëkke* (eldest sister-in-law, maybe two), – all of whom likely live under the same roof – and maybe some others, like their *bajen* (their own father’s eldest sister), their co-wives (if applicable), or a very close friend (see Fig. 1). As discussed above, pregnancy is a taboo subject and is therefore not to be discussed with or even shown to anyone beyond that inner circle for fear that bad luck or misfortune could befall the pregnancy, the pregnant woman, the unborn fetus, or the newborn child. The only exception that is usually made is for healthcare providers (e.g., midwives, physicians) and state-trained neighborhood birth workers (called *bajenu gox* [“neighborhood aunts” in English]). Women often regard birth workers like midwives and *bajenu gox* as honorary members of their entourage.

The entourage acts similarly to what the medical anthropologist John Janzen identified as a “therapy management group” (TMG) [23]. Therapy management, Janzen highlights, involves diagnosis, selection, and evaluation of treatment, as well as support of the sufferer, while the therapy management *group* is a group of family or friends which emerges around a sufferer to take charge of those responsibilities. An entourage, on the other hand, emerges as a kin-based collective that provides emotional support, physical safety, and financial security. Whereas the TMG typically acts on behalf of incapacitated patients, the entourage does not view pregnant women as incapable of making decisions for themselves. She is not considered a “sufferer,” nor is pregnancy considered an “illness.” They are there to help her navigate her experiences with pregnancy and in biomedical spaces, such as maternity wards.

The responsibilities of antenatal care are distributed to individuals in the entourage, therefore no one person is wholly responsible for her care. Some tasks can be shared by multiple people in the entourage (e.g., assuming her domestic tasks, like cooking and laundry), while some are the sole responsibility of one individual (e.g., intimate care). The goro (mother-in-law), for example, is responsible for sharing intergenerational obstetric knowledge, such that an entourage may necessarily become more hands-off with each successive pregnancy (so long as previous pregnancies have had consistently positive outcomes). She is like the project manager, while the njëkke is her second-in-command. The njëkke (the sister-in-law) is a kind of organizer who both works with the goro to execute certain tasks or preparations while also learning obstetric knowledge from her as well (if she is inexperienced). She is also responsible for naming the newborn at the *ngente* (naming ceremony) that takes place seven days after the child is born. Either the goro or the njëkke (or both) can accompany the pregnant woman to antenatal care (ANC) visits. I will discuss expectant fathers’ roles next.

### What men are expected to do

The entourage has two specific expectations of expectant fathers: that they demonstrate (1) financial and (2) emotional support.

#### Financial support

Financial support, called *sersé larzã* (“find the money” in Dakarois French), is, according to the midwives and goros in this study, the most important job of anyone in the entourage. Wages are low and employment can be difficult to find, so for the working-class men who participated in this research (and who make up the vast majority of the Senegalese population), finding money consumes a great deal of time and effort. It can involve working multiple jobs and one-offs, often in dangerous manual labor (with little or no worker protections), as well as calling expatriated relatives in Europe or North America to seek financial assistance [24].

During my research, I observed that financial support funds all manner of expenses during pregnancy. It buys taxi fare to take pregnant women to their ANC visits so that she doesn’t have to inhale the diesel fumes of the city busses. It buys her a consultation ticket for those exams and pays for her prescriptions and vitamins. It puts food on the table and keeps the lights on. It pays the woman down the street to come and do the laundry and clean the house so that pregnant women in their second and third trimesters don’t have to. It buys the ram (about USD$500) which will be sacrificed at the ngente (naming ceremony) seven days after the child. Perhaps most bleakly, it pays for the multiple taxi fares and consultation tickets on the day of the delivery because she will undoubtedly be told to go from hospital to hospital while she is in labor. Indeed, all of the participants in my study visited two to four hospitals while they were in labor before they gave birth. Financial support pays the hospital bill and, if necessary, it bribes the right people to put down their cellphones and do their jobs. If there is ever a surplus of money, it will buy her little gifts and special treats throughout the pregnancy so that he can keep her in good spirits and reassure her that she is not alone. Financial support overlaps with emotional support, as women report feeling more safe, secure, and attended to when their partners demonstrated financial support.

#### Emotional support

Midwives also acknowledge that not all men can pay for all of these expenses and that the inability to demonstrate consistent financial support does not make one “uninvolved.” There are other non-financial forms of emotional support that can keep her in good spirits. Emotional support can take many forms, although who can undertake those forms is often determined by relationship to the pregnant woman as well as the gender of the caring person. In the case of expectant fathers, popular forms of emotional support are to look out for her wellbeing by enforcing certain behavioral and nutritional prescriptions and proscriptions given in the clinics, making sure she is taking any medications she may need, and reminding her of her clinical appointments. Some women reported that they appreciated when their husbands called them multiple times a day to check in, viewing it as a demonstration of care and interest in her health and wellbeing. Women also felt positively about men who acted as ad hoc health interpreters for them. It was frequently the case that, as a result of gendered disparities in education, men were more literate than their pregnant partners. This produces a dynamic in which expectant fathers could provide emotional support by reading educational materials about pregnancy, childbirth, and motherhood for her.

### What men are not expected to do

The expectations of the entourage are often determined by the gender of the entourage member and their relationship to the pregnant woman. As discussed above, pregnancy is highly gendered in Senegal and known as a form of what is called *affaire-u-jiggeen*, or “women’s business” (in Wolofrançais). Men are not only *not expected* to get involved in the same ways that women are involved, but they can also be actively shut out from certain activities. Expectant fathers’ mothers (i.e., the goro) are the senior women of the entourage and therefore call the shots (although there are some interesting political frictions that can emerge between her and her daughters that fall beyond the scope of this article). For example, these mothers may either aggressively bar their sons from attending ANC visits, or passive-aggressively question their sanity, masculinity, or African-ness for even suggesting they may attend. On this latter point, overly active expectant fathers are sometimes teased for being like “Europeans” or “white people.” The domain of “women’s business” is not unilaterally constructed by men as a way of avoiding effeminate activities, rather it is co-constructed by women as a matter of maintaining their autonomy over tasks considered to be within their domain [25]. It protects not only pregnancy, but domestic labor and meal preparation as well.

This should shed some light on the question of why men don’t attend ANC visits or talk to healthcare providers about family planning, as Assaf and Davis found. First, as discussed, men are busy trying to find money. Maternity wards are often crowded and waiting can last hours (even if there was an appointment). Families cannot afford for men to take off that amount of time when every income generating activity counts. Second, men are socially barred from attending. It is critical to understand that just because he doesn’t go with her, that doesn’t mean she goes alone. This is the purpose of the entourage system of distributed care responsibilities.

Over hundreds of hours spent in maternity wards, I have seen no more than ten men in the waiting room. In interviews with these rare men who *do* attend ANC visits, they say they often don’t feel welcome and are sometimes actively made to feel unwelcome. The men who attend ANC visits tend to be better educated and with more stable employment. They can afford to take the time off and they’re actively interested in what healthcare professionals have to say. It is far more common for men to be present during labor and delivery, insofar as sitting in the waiting room is “present.” These men have reported that they have less than ideal interactions with junior midwives (who make up the vast majority of the midwifery workforce) who they described as “never getting off their phones” or “actively ignoring me.” It was common, in all of the births I attended, to check in with men about the progress of labor only to hear, “I don’t know. The nurses won’t tell me anything.” In one case, while I was present with a family of a laboring woman at a large public hospital, the father and I were asked by a midwife to deliver blood samples to the lab across the campus and return with results. When we received the results, the father didn’t know what they meant, but I was able to explain on the way back to the midwife. When he asked the midwife to explain the test results, she simply said, “You wouldn’t understand.”

## Discussion

In his critical discourse analysis of a USAID *Epidemiological Profile of El Salvador* [26], Luis Avilés writes,

“These implicit rules [of discourse] are based on the notion that knowledge and power implicate each other. The exercise of power requires the production of certain types of knowledge, while the production of knowledge requires the exercise of power to validate its assertions” [16].

The implicit rules of discourse, knowledge, and power are interlaced into the design and execution of DHS as well as the limitations it places upon the possibilities for operational definitions in reports like DHS 64. Discourse is also mobilized when the inherent values of such a report are reproduced by its dissemination. In DHS 64, the purportedly *global* values placed on family are heterosexist, monogamous, nuclear, and fully dependent on a specific set of gender stereotypes that make no room for the generative and creative possibilities of non-European networks of kinship and care. Avilés writes of the Eurocentricity that is deeply interwoven into development discourse such that, simply put, “Europe is the model to emulate” [16]. So too do such globalized formulations, not just of family but of masculinity, perpetuate longstanding deficit-based discourses of Black and Brown men in LMIC [27, 28]. Furthermore, they ultimately do nothing to address or bring attention to the historical and political determinants of women’s and children’s health [29].

USAID’s characterization of men as “uninvolved” was a surprising revelation to the Senegalese physicians, midwives, mothers, fathers, and Ministry of Health officials in this study who unanimously viewed men as an integral part of women’s and children’s health. It was apparent to many that the authors of DHS 64 had never been to Senegal to examine the topic of their report. One physician remarked that USAID could not see the deep level of men’s engagement that I was seeing. One midwife noted with irony that there were men who went above and beyond and wished they could do even more for their pregnant partners. The key findings of this article demonstrate why Assaf and Davis’ operationalization of “men’s involvement” is not sufficient for understanding the full picture of how (or whether) Senegalese men are involved in contributing support and care for women’s and children’s health. Further, without a holistic view of what Senegalese men are expected to do or are actually doing, there can therefore be no reliable analysis of whether local expectations have a positive effect on health outcomes.

I speculate that an intervention based on findings like those in DHS 64 would have harmful downstream effects on both women’s and children’s health, as well as future public health research and practice. Such interventions would likely fail by attempting to address issues that are not only *not* viewed as problems locally, but are accepted as assets of local gender and kin norms. As Lucia Guerra-Reyes’ work [30] demonstrates, global health interventions that ignore local contexts in favor of culturally inappropriate global metrics can be a source of physical and emotional coercion of pregnant women and their families, as well as overwork or redundancy for care providers, all in an effort to meet project goals. Such interventions, frustratingly, would also fail to address the issues that local stakeholders agree are harmful determinants of women’s and children’s health, such as a lack of hospital resources and fair wages for staff. These political determinants, as Avilés writes, are beyond the purview of development organizations like USAID [16], and it therefore makes sense why the authors are disincentivized from considering historical contexts of poor health. This study reveals an urgent need (though by no means new [31–33]) for global and public health engagement with local stakeholders and ethnographic researchers who can finely-tune context-specific operational definitions and program objectives and goals in line with local needs.

## Conclusion

As it relates directly to this study, Assaf and Davis report in DHS 64 that men are not likely to be present during ANC visits or discuss family planning with a healthcare worker. While my ethnographic data support both of these findings, they do not support the implicit conclusions of such global health logics that Senegalese men are therefore uninvolved in women and children’s health. Senegalese men are involved in women and children’s health in ways that the DHS does not track. The numbers simply do not tell the whole story.

In this article, I have demonstrated that in Senegal, antenatal care roles and responsibilities are distributed among a small network of kin-relations called the entourage. As a part of this entourage, expectant fathers are relied upon to find money to pay for the added expenses of pregnancy and to provide emotional support – often the two overlap. Pregnant women, their mothers-in-law, and midwives all agree that these responsibilities are critical to the health and wellbeing of women and children, and yet almost none of them serve as indicators of “men’s involvement” in global health literature, nor can they be measured by current indicators in the DHS.

I have also demonstrated that Senegalese men do not undertake the forms of care and support that Assaf and Davis call “involvement” because those responsibilities are not theirs to act upon, nor would it be financially feasible for most Senegalese.

Finally, I have demonstrated that Eurocentric measures of men’s involvement, while ostensibly universal, are so narrow as to be impractical for intervention. The assumption that, because men aren’t accompanying their pregnant partners to ANC visits, no one is accompanying her reveals deeply entrenched ethnocentric and limited conceptions of masculinity, of family dynamics, and of care. Narrow conceptions of what a father “should” know or do as forms of maternal support are also tied to equally myopic assumptions of nuclear households that presume men are solely responsible for women’s and children’s care. These measures tell us nothing about the relationship between men’s actions and women and children’s health when men are not the only people involved in their care. Like “culture,” kinship is not fixed nor bounded—even in nuclear families (which is a rare occurrence in Dakar). It is important to widen the scope of interrogation to include all meaningful relationships in someone’s life, perhaps approximating what I’ve described here as an entourage or Janzen’s TMG. Measures of men’s involvement can only be meaningful in locally specific, context dependent analysis and practice.

## Supplementary Information

**Additional file 1. **Semi-structured interview guide for pregnant women.

**Additional file 2. **Semi-structured interview guide for expectant fathers.

## Data Availability

English translations of interview guides are provided as supplementary files to this article. Original French interview guides are available from the corresponding author on reasonable request. The datasets generated and/or analysed during the current study are in French and Wolof and are not publicly available due to confidentiality of the participants but will be considered from the corresponding author on case-by-case basis.
