# Supplementary material for: (Mis)measuring men’s involvement in global health: the case of expectant fathers in Dakar, Senegal
Source: BMC Pregnancy Childbirth. 2022 Oct 7;22:754. doi: 10.1186/s12884-022-05093-0 (PMC9541010; doi:10.1186/s12884-022-05093-0)
Supplement: Supplementary file 1 — Additional file 1. Semi-structured interview guide for pregnant women. [file 12884_2022_5093_MOESM1_ESM.pdf]

ROR2018 Interview Guide, Pregnant Women (*TRANSLATED FROM FRENCH*)

Archive # :
Interviewer :

☐ Introduction

I am a researcher working with a team on a project about pregnancy and men in Dakar.

Now, we're conducting interviews to better understand how people take care of pregnant women and how expectant fathers are involved in that care. This interview is like a discussion. What we want to learn is your personal experience and what you think of these subjects. I have specific questions, but I invite you to speak about what you think is important.

- Briefly describe the study (see above)
- Read confidentiality statement and obtain clear consent for the interview and recording
- Make sure the recorder is on and functioning correctly
- Confirm consent again after the recording begins

☐ Do you have any questions before we begin ?

Heure :

GENERAL

☐ **Question 1**. First, I'd like to know about you. Talk to me about you and your family.

➢ **Probe** : [Non-specific]

➢ **Probe** :

☐ **Childhood** – Where did you grow up?

☐ **Time** – How long have you lived in Dakar?

☐ **Social relations** – Can you tell me about your family and friends?

*Archive # : END[initiales d'interviewer][chiffre] (par exemple : ENDDP01)

☐ **Question 2**. I would like for you to tell me about your typical day. What do you do from the time you wake up to the time you go to bed?

- **Probe**: [Non-specific]

- **Probe** :

  ☐ **Examples** – School, work, chores, kids, friends

  ☐ **Pleasure** – What do you do for fun?

Experience with Pregnancy

☐ **Question 3**. Now I would like you to talk to me about your experience with pregnancy. Could you tell me about when you found out you were pregnant?

- **Probe**: [Non-specific]

- **Probe**:

  ☐ **When** – At what point in the pregnancy? What date?

  ☐ **How** – Did you think you might be pregnant before the test? Why?

  ☐ **Why** – Why did you get a pregnancy test?

  ☐ **Who** – Who did you tell when you found out? What was their response?

  ☐ **Partner**: When did you tell your partner? How did he respond?

☐ **Question 4** : How have you adapted to being pregnant?

- **Probe**: [Non-specific]

- **Probe**:

  ☐ **Behavior** – How must a pregnant woman behave for her own health and the health of her child?

  ☐ **Clothing** – What kind of clothing is best for a pregnant woman?

  ☐ **Health** – What kind of food and physical efforts are necessary for a pregnant woman?

  ☐ **Visits** – Do you go to all of your CPNs and follow the instructions of the midwife? Why?

☐ **Question 5** : I am curious to know how the pregnancy has become evident in your daily life. Can you tell who you talk to about it and what you talk about?

- **Probe**: [Non-specific]

- **Probe**:

  ☐ **Advice** – Who do you go to for advice?

  ☐ **Where** – Where do you have these conversations? Why?

  ☐ **Partners** – Is there anything about the pregnancy that you don't talk to your partner about? Why?

☐ **Question 6** : Is it okay if we talk about bad luck? I would like to know how pregnant women avoid bad luck and how their families help them.

- **Probe**: [Non-specific]

- **Probe**:

  ☐ **How** – How does bad luck show up?

  ☐ **Society** – What types of things cause bad luck?

  ☐ **How** – What are the things that a pregnant woman and her family can do to avoid bad luck?

  ☐ **Marabout** – Have you consulted a marabout? What does he do to help protect you?

MEN'S INVOLVEMENT

☐ **Question 7** : Could you enumerate all of the examples of all of the ways what someone can take care of a pregnant woman?

☐ **Question 8** : What are some ways that people take care of you?

- **Probe** : [Non-specific]

- **Probe** :

  ☐ **Support** – What kind of psychological, emotional, and moral support do pregnant women need from their partners and family?

☐ **Question 9** : What are the ways that your partner takes care of you?

- **Probe** : [Non-specific]

- **Probe**:

  ☐ **Changes** – Do you think that men change during pregnancy? Has your partner changed? How?

  ☐ **Behavior** – How should a partner behave toward his pregnant wife? Is this the case in your own situation?

  ☐ **Pet peeves** – What is something he does that bugs you?

  ☐ **Gestures** – What are the little things he does that make you feel pleasure?

☐ **Question 10** : What things would you like your partner to do that he does not?

- **Probe** : [Non-specific]

- **Probe** :

  ☐ **Why** – Why doesn't he do these things?

☐ Demographic Information

1. Age : ________(years)
2. Gender : ○1 Man ○2 Woman
3. Marital Status : ○1 Single ○2 Engaged ○3 Married ○4 Divorced ○5 Widowed
   - [If not single] : How many wives/co-spouses? ______
   - [If a married woman with co-spouses] : Which number co-spouse are you? ______
4. Do you have children? Boys: ______ Girls: ______
5. Imagine yourself on a ladder from 1 to 10 representing the people of Dakar. At the top are the 10s, people who have the most money, the best education, and the best jobs. At the bottom are the 1s, the people with the least money, the least education, and the least respected jobs.

   Choosing a number from 1 to 10, where do you find yourself on this ladder? ______

6. What is the highest level of education you have?
   - ○ 1 Ecole coranique/moins que lycée
   - ○ 2 Lycée/BAC
   - ○ 3 License ou License Professionnelle
   - ○ 4 Master 1/2 (MA, MS, MPhil, etc.)
   - ○ 5 Doctorat (PhD, EdD, DPhil, etc.)
   - ○ 6 Professionnel (MD, JD, DDS, etc.)

7. Which of these best describes your daily activities and responsibilities? (Choose all that are applicable.)

- ○ 1 I work all the time.
- ○ 2 I work some of the time.
- ○ 3 I am unemployed.
- ○ 4 I am looking for a job.
- ○ 5 I clean the house and raise the children.
- ○ 6 I am retired.

8. What do you do (or did you do) for work?

The End

☐ These are all the questions we have for you. Are there other things or experiences that you would like to share or is there something we didn't ask that you think is we should know?

Could you refer us to your partner for an interview?

☐ We would like to talk to your partner too. Everything will be the same – an interview for one hour for 2.000fcfa – except the questions will be a little different. As we mentioned, we promise to keep your responses confidential, and we will not share anything you told us with him. I assure it.

[tear away contact information for follow-up or chain-referral]

ROR2018 – END – FE (FR) 7 sur 9 Archive #: __________
