# Supplementary material for: (Mis)measuring men’s involvement in global health: the case of expectant fathers in Dakar, Senegal
Source: BMC Pregnancy Childbirth. 2022 Oct 7;22:754. doi: 10.1186/s12884-022-05093-0 (PMC9541010; doi:10.1186/s12884-022-05093-0)
Supplement: Supplementary file 2 — Additional file 2. Semi-structured interview guide for expectant fathers. [file 12884_2022_5093_MOESM2_ESM.pdf]

ROR2018 Interview Guide, Expectant Fathers (*TRANSLATED FROM FRENCH*)

Archive # :
Interviewer :

☐ Introduction

I am a researcher working with a team on a project about pregnancy and men in Dakar.

Now, we're conducting interviews to better understand how people take care of pregnant women and how expectant fathers are involved in that care. This interview is like a discussion. What we want to learn is your personal experience and what you think of these subjects. I have specific questions, but I invite you to speak about what you think is important.

- Briefly describe the study (see above)
- Read confidentiality statement and obtain clear consent for the interview and recording
- Make sure the recorder is on and functioning correctly
- Confirm consent again after the recording begins

☐ Do you have any questions before we begin ?

Heure :

GENERAL

☐ **Question 1**. First, I'd like to know about you. Talk to me about you and your family.

- ➢ **Probe** : [Non-specific]

- ➢ **Probe** :
  - ☐ **Childhood** – Where did you grow up?
  - ☐ **Time** – How long have you lived in Dakar?
  - ☐ **Social relations** – Can you tell me about your family and friends?

*Archive # : END[initiales d'interviewer][chiffre] (par exemple : ENDDP01)

☐ **Question 2**. I would like for you to tell me about your typical day. What do you do from the time you wake up to the time you go to bed?

- **Probe**: [Non-specific]

- **Probe** :

  ☐ **Examples** – School, work, chores, kids, friends

  ☐ **Pleasure** – What do you do for fun?

EXPERIENCE WITH PRENGNANCY

☐ **Question 3**. Now I would like you to talk to me about your experience with pregnancy. Could you tell me about when you found out your partner was pregnant?

- **Probe**: [Non-specific]

- **Sondage** :

  ☐ **When** – At what point in the pregnancy? What date?

  ☐ **How** – What did you think when she told you she was pregnant?

  ☐ **Why** – Why did she seek a test?

  ☐ **Who** – Who did she tell when she discovered? How did they respond?

  ☐ **Partner** – When did you partner tell you? Could you describe for us your emotions when she first told you?

  ☐ **Who** – Who have you told? How did they respond?

☐ **Question 4** : How has she adapted to the pregnancy?

- **Probe**: [Non-specific]

- **Probe**:

  ☐ **Behavior** – How must a pregnant woman behave for her own health and the health of her child?

  ☐ **Clothing** – What kind of clothing is best for a pregnant woman?

  ☐ **Health** – What kind of food and physical efforts are necessary for a pregnant woman?

  ☐ **Visits** – Does she go to all of her CPNs and follow the instructions of the midwife? Why?

MEN'S INVOLVEMENT

☐ **Question 5** : I am curious to know how the pregnancy has become evident in your daily life. In what ways have you adapted to the pregnancy?

- **Probe**: [Non-specific]

- **Sondage** :

  ☐ **Behaviors** – Your daily routine

  ☐ **Thoughts** – Your way of thinking

  ☐ **Network** – Could you tell me about who you talk to about it and what you talk about?

☐ **Question 6** : Could you enumerate all of the examples of all of the ways what someone can take care of a pregnant woman?

☐**Question 7** : What role do you play during the pregnancy of your partner?

- **Probe** : [Non-specific]

- **Sondage** :

  ☐**Money** – What do you do to secure yourself financially?

  ☐**Hospital** – How do you encourage your wife to keep up with her CPNs?

  ☐**Emotional support** – What psychological support is most appropriate for your wife?

  ☐**Physical Support** – How do you physically support your pregnant wife?

  ☐**Negatives** – What do you do to/for your pregnant wife that you probably shouldn't?

  ☐**Mother-in-law** – According to you, is it normal to send your pregnant wife to her mother's house when she is pregnant?

  ☐**[If yes]** – What can she do for your partner that you cannot?

☐ **Question 8** : Can you tell me who you talk to about these things and what you talk about?

- **Probe** : [Non-specific]

- **Sondage** :

  ☐ **Others** – Who do you not talk to about these things? Why not?

  ☐ **Taboo** – Pregnancy is taboo. Does talking about it bring bad luck and why/how?

☐ Demographic Information

1. Age : ________(years)
2. Gender : ○ 1 Man ○ 2 Woman
3. Marital Status : ○ 1 Single ○ 2 Engaged ○ 3 Married ○ 4 Divorced ○ 5 Widowed
   - [If not single] : How many wives/co-spouses? ______
   - [If a married woman with co-spouses] : Which number co-spouse are you? ______
4. Do you have children? Boys: ______ Girls: ______
5. Imagine yourself on a ladder from 1 to 10 representing the people of Dakar. At the top are the 10s, people who have the most money, the best education, and the best jobs. At the bottom are the 1s, the people with the least money, the least education, and the least respected jobs.

   Choosing a number from 1 to 10, where do you find yourself on this ladder? ______

6. What is the highest level of education you have?
   - ○ 1 Ecole coranique/moins que lycée
   - ○ 2 Lycée/BAC
   - ○ 3 License ou License Professionnelle
   - ○ 4 Master 1/2 (MA, MS, MPhil, etc.)
   - ○ 5 Doctorat (PhD, EdD, DPhil, etc.)
   - ○ 6 Professionnel (MD, JD, DDS, etc.)

7. Which of these best describes your daily activities and responsibilities? (Choose all that are applicable.)

- ○ 1 I work all the time.
- ○ 2 I work some of the time.
- ○ 3 I am unemployed.
- ○ 4 I am looking for a job.
- ○ 5 I clean the house and raise the children.
- ○ 6 I am retired.

8. What do you do (or did you do) for work?

☐ The End

These are all the questions we have for you. Are there other things or experiences that you would like to share or is there something we didn't ask that you think is we should know?

☐ Could you refer us to two friends or family members for interviews?

We would like to talk to two close friends/family: someone from whom you seek advice about pregnancy and fatherhood. As we mentioned, we promise to keep your responses confidential, and we will not share anything you told us with him. I assure it.

[tear away contact information for follow-up or chain-referral]	[tear away contact information for follow-up or chain-referral]
